# Aberrant methylation and microRNA-target regulation are associated with downregulated NEURL1B: a diagnostic and prognostic target in colon cancer

**DOI:** 10.1186/s12935-020-01379-5

**Published:** 2020-07-27

**Authors:** Jiaxin Liu, Zhao Liu, Xiaozhi Zhang, Yanli Yan, Shuai Shao, Demao Yao, Tuotuo Gong

**Affiliations:** 1 Department of Geriatric Surgery, The First Affiliated Hospital of Xi’an Jiaotong University, Xi’an, 710061 The People’s Republic of China; 2 Department of Oncology Surgery, The First Affiliated Hospital of Xi’an Jiaotong University, Xi’an, 710061 The People’s Republic of China; 3 Department of Radiotherapy, The First Affiliated Hospital of Xi’an Jiaotong University, Xi’an, 710061 The People’s Republic of China

**Keywords:** Colon cancer, Methylation, Bioinformatic analysis, Early diagnosis and prognosis, microRNA

## Abstract

**Background:**

Aberrant methylation and miRNA-target-gene regulation function as important mechanisms for gene inactivation in colon carcinogenesis. Although a serious of molecular events (such as aberrant alterations of genomics and epigenetics) have been identified to be related to prognostic in colon cancer (CC) patients, beneficial biomarkers for early diagnosis and prognostic evaluation remain largely unknown.

**Methods:**

In our study, the role of NEURL1B, including gene expression analysis, methylation characteristic, miRNA-target regulation, diagnostic and prognostic significance, were evaculated using multiple bioinformatic tools based on TCGA database and clinical samples.

**Results:**

Our data showed that NEURL1B was aberrantly downregulated in CC, regardless of the mRNA level or protein level. Moreover, ROC curve and multivariate Cox regression analysis demonstrated that NEURL1B was a diagnostic and independent prognostic facter for CC patients. Of interest, methylation of NEURL1B was also high and closely associated with poor survival in CC. In addition, multiple NEURL1B-target miRNAs were found to be overexpressed in CC tissues. Thus, our findings suggested that NEURL1B participated in the pathological processes of CC as a tumor suppressor gene. Double management, including DNA methylation modification and miRNA-target regulation, were considered to be related to the downregulation of NEURL1B. Importantly, there existing be an significant intersection between miRNAs-target pathways and NEURL1B-target pathways, suggesting that miR-17 and miR-27a might promote tumor cell malignant property by targeting NEURL1B degradation via the activation of PI3K/AKT signaling pathway.

**Conclusions:**

Taking together, the first investigation of NEURL1B in CC provide us a strong evidences that it might be served as a potential biomarkers for early diagnosis and prognostic evaluation in CC.

## Background

Colon cancer (CC) is one of the most common malignant tumors of the digestive system in recent years around the world. According to the data from the Centers for Disease Control and Prevention’s (CDC’s) National Center for Health Statistics (NCHS), there were 135,430 new CC cases and 50,260 deaths in 2017 in the United States [1], however, an estimated 376.3 per 100,000 new CC cases and 191.0 per 100,000 cancer deaths occured in China in 2015 through the National Central Cancer Registry of China [2]. For patients with CC, early diagnosis and surgical resection are currently the standard treatment [3], unfortunately most of patients were detected at a late stage and poor prognosis was predicted due to losing optimal timing of surgery or only undergoing palliative surgery [4]. Therefore, it is urgent to identify early diagnostic and prognostic biomarkers for CC.

Tumors are currently considered to be a genetic and epigenetic disease. Epigenetic changes play a more important role in the initiation and progression of tumors than genetics and may occur prior to genetic changes [5]. As an important epigenetic event, more and more evidences show DNA methylation is the most extensive and in-depth epigenetic mechanism. In human cancers, the hypomethylation of the entire gene sequence is the earliest epigenetic change and hypermethylation of the tumor suppressor gene in promoter region leads to gene inactivation, which is considered to be an important mechanism of tumorigenesis [6–10]. Human Neuralized 2 (NEURL1B), a crucial gene, is dispensable during embryonic development. Its expression is high in several peripheral tissues including heart, liver and testis [11]. But limited studies are focused on CC, whose function and role in CC remain unclear.

In the present study, we firstly assessed the expression and prognosis of NEURL1B. Then, methylation characteristic and miRNA-target regulation were analysed to identified downregulated mechanism of NEURL1B in CC. Finally, diagnostic capability was evaluated based on a ROC curve. In conclusion, the first investigation of NEURL1B in CC provide us a strong evidences that it might be served as a potential biomarkers for early diagnosis and prognostic evaluation in CC.

## Methods

### Microarray data analysis

Three gene expression profiles, including GSE64658 (GPL1261), GSE44904 (GPL7202) and GSE31106 (GPL1261), were selected from the Gene Expression Omnibus (GEO, https://www.ncbi.nlm.nih.gov/) database [12]. Each profile, including colon cancer (CC) and normal colon (NC) tissues, was analysed using GEO2R (http://www.ncbi.nlm.nih.gov/geo/geo2r/) online tool [13] and thousands of differentially expressed genes (DEGs) were visible.

### NEURL1B filtering

DEGs were filtered according to the fold change (FC) and adjusted P values (adj. P). 5329 DEGs, followed by 3176 and 481 DEGs were respectively in sight in GSE44904, GSE64658 and GSE31106. Then the Online Omicshare 3.0 (http://www.omicshare.com/tools) was performed to discover the overlapping genes among different profiles. Three-crossing or two-crossing genes were screened on the basis of multiple criterions: a. There was a significant difference for the expression of DEGs between CC and NC tissues from TCGA database. b. OncoLnc online analysis (http://www.oncolnc.org/) [14] revealed an association between expression of DEGs and the prognosis of CC, and P < 0.05 was set as the cut-off value.

### Expression analysis of NEURL1B

A comprehensive investigation based on TCGA database and the Human Protein Atlas (THPA, https://www.proteinatlas.org/) tool, as well as 13 pairs of clinical samples from the first affiliated hospital of Xi’an Jiaotong university from April 2018 to November 2018, was performed to evaluate expression of NEURL1B in the mRNA and protein level. RNA was extracted and reversed to cDNA, qRT-PCR was performed as previously described [15]. The primers used are as follow: NEURL1B: forward: 5′-CCA AAG GCA AGA ACG TGC GG-3′, reverse: 5′-GTA CTC TTT GCG GTC GAG CA-3′; β-actin: forward: 5′-CCT TGC ACA TGC CGG AG-3′, reverse: 5′-GCA CAG AGC CTC GCC TT-3′. Besides, tissues were also fixed in 4% formaldehyde at room temperature for 48 h in preparation for immunohistochemistry (IHC) with rabbit anti-NEURL1B primary antibody (ab122400) in PBS (1:300) overnight at 4 °C as previously described [15]. In addition, χ^2^-test from TCGA database was used to analysis the association between NEURL1B expression and clinicopathological variables in CC patients, including age, gender, tumor stage, lymph metastasis, distant metastasis and clinical stage.

### Prognostic analysis of NEURL1B

NEURL1B was submitted to OncoLnc, a prognostic analysis tool for multiple tumors, to reveal the correlation between its expression and overall survival (OS). To determine whether the prognostic significance of NEURL1B was independent of the above-mentioned clinicopathological variables in CC, univariate and multivariate Cox regression analysis were performed.

### Methylation analysis of NEURL1B

We assessed the methylation of NEURL1B using multiple methods. MethHC (http://methhc.mbc.nctu.edu.tw/php/index.php), a web based resource focused on the DNA methylation of human diseases that includes 18 human cancers over 6000 samples and 6548 microarray and 12,567 RNA sequencing data [16], was firstly used to detect methylation situation. ^**^: P < 0.005; ^*^: P < 0.05. In addition, UALCAN (http://ualcan.path.uab.edu/), which could provide promoter DNA methylation data from the TCGA Infinium Human Methylation 450 K Bead Chip arrays for most of the genes & TCGA cancer types [17], was also performed to analysis promoter methylation level. MethSurv (https://biit.cs.ut.ee/methsurv/), the third online way, a web tool to perform multivariable survival analysis using DNA methylation data [18], was executed to assess different CpG islands scattering. Importantly, we also explored the expression of 3 DNA methyltransferases (DNMT1, DNMT3A and DNMT3B) between NEURL1B^High^ and NEURL1B^Low^ based on TCGA database.

### Clinical value of NEURL1B methylation

To further explore the clinical role of NEURL1B methylation in CC, UALCAN was performed to clarify the relationship between NEURL1B methylation and clinical elements that included age, gender, race, weight and stages. Besides, MethSurv also contributed to the connection between position distribution around CpG islands and prognosis of CC patients.

### Pathways and protein–protein interaction analysis

GSCALite (http://bioinfo.life.hust.edu.cn/web/GSCALite/), which is a web-based analysis platform for gene set cancer analysis and could contribute to the cancer initiation, progress, diagnosis, prognosis, therapy [19], was available to predict the signaling pathways involved in CC. Among the activated pathways, some critical molecules were picked up, Spearman’s or Pearson’s correlation was analysed via cBioPortal (https://www.cbioportal.org/), which provided visualization and analysis of large-scale cancer genomics data sets [20]. In addition, protein-protein interaction (PPI) analysis from STRING (http://string-db.org/cgi/input.pl) [21] was input Cytoscape software to illustrate integrated protein. Simultaneously, a permutatio test p-value of NEURL1B expression between driver mutated and non-mutated samples were examined using TCGAportal (www.tcgaportal.org).

### miRNA regulation of NEURL1B

Multiple tools, including miRANDA, miCODE, miRDB, miRWalK, miTarbase and TargetScan, were used to predict the NEURL1B-targeted miRNAs. The overlapping miRNAs were additionally filtered using Omicshare. Then, we detected the expression of different NEURL1B-targeted miRNAs on the basis of TCGA database by GraphPad software. In addition, miRNAs-dependent signaling pathways and GO analysis were also evaluated via ONCOMIR (http://www.oncomir.org/) [22].

### Diagnostic value of NEURL1B

A reciever operating characteristic (ROC) curve was used to analyse the diagnostic ability of NEURL1B in CC. In addition, To further compare the diagnostic ability between NEURL1B and clinically common biomarkers, carcinoembryonic antigen (CEA) was also applied to react the difference.

### Statistical analysis

All data were analysed using the SPSS statistical package (version17.0; SPSS Inc., Chicago, IL, USA). The association between NEURL1B expression and clinicopathological characteristics were evaluated by χ^2^ tests and Spearman’s correlation analysis. Univariate and multivariate analysis were based on Cox proportional hazard regression models. Student’s t-test was performed to compare the expression of Neurl1b between CC and NC tissues. P < 0.05 was considered to indicate a statistically significant difference.

## Results

### Gene expression profiles analysis and DEGs filtering

**Fig. 1:**
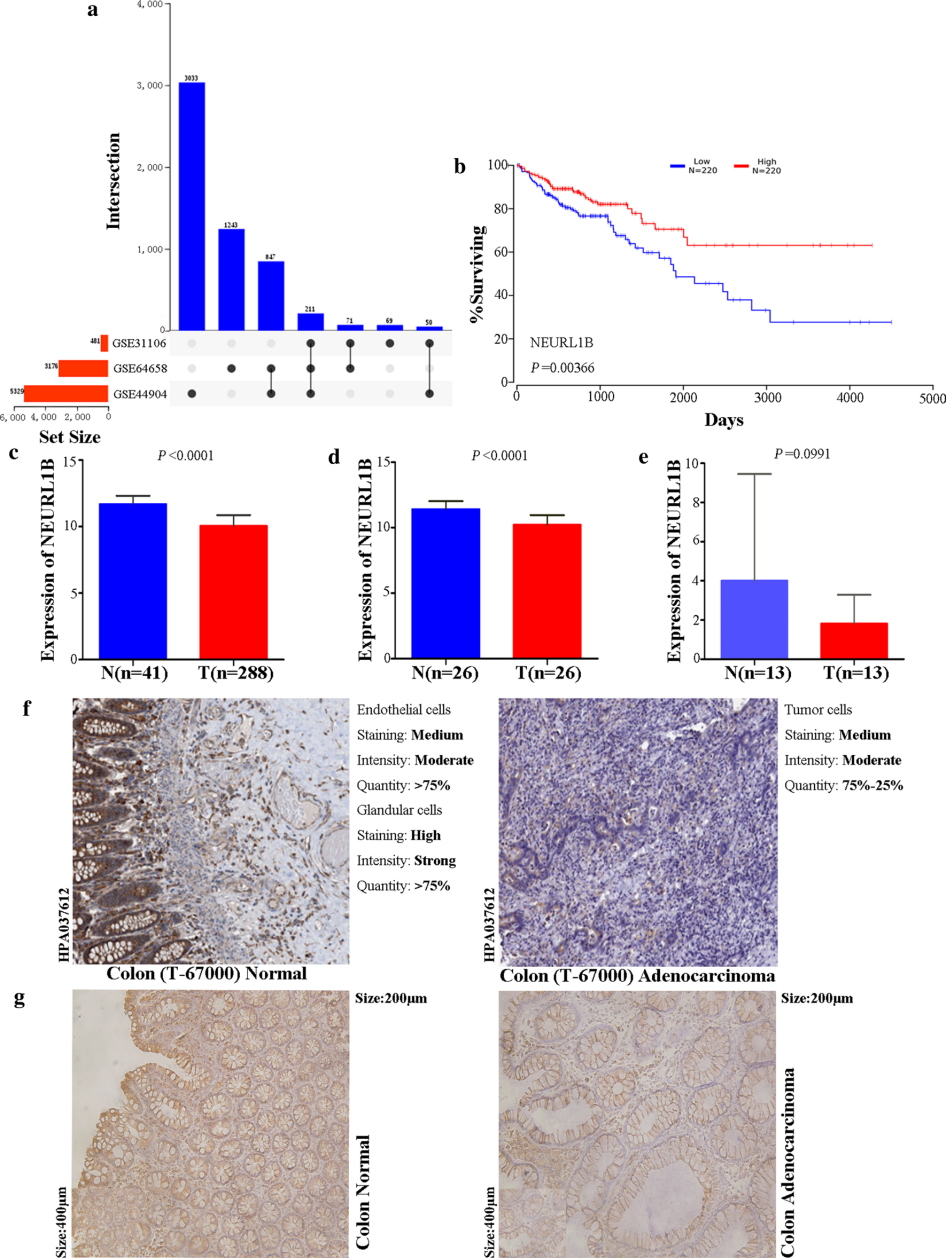
Filtering of NEURL1B and its clinical value. **a** DEGs filtering based on microarray data analysis. **b** Prognostic significance of NEURL1B in CC based on OncoLnc analysis. **c**, **d**. mRNA expression of NEURL1B based on TCGA database, including 41 NC and 288 CC tissues,as well as 26 paired of NC and CC tissues was analysed. **e** mRNA expression of NEURL1B based on 13 pairs of clinical samples. **f**, **g**. Immunohistochemistry detection based on THPA tool and 13 pairs of clinical samples

A total of 211 three-crossing and 968 two-crossing genes from Omicshare online tool (847 between GSE64658 and GSE44904, 71 between GSE64658 and GSE31106, 50 between GSE44904 and GSE31106) were filtered (Fig. 1a). Combined with prognostic value (Fig. 1b), NEURL1B was selected in the present study.

### High expression of NEURL1B in NC

Comparing mRNA expression data based on the TCGA database demonstrated significant differences in the expression of NEURL1B between NC and CC tissues (Fig. 1c, d). However, we obtained a different result when 13 matched pairs of clinical samples were compared (P = 0.0991, Fig. 1e). Besides, protein expression analysis from THPA tool and clinical samples also showed NEURL1B was overexpressed in colon endothelial cells and glandular cells than colon tumor cells (Fig. 1f, g). In addition, some clinicopathological variables, including age, tumor stage, lymph metastasis and clinical stage, were negatively associated with NEURL1B expression using χ^2^-test analysis (Table 1).

**Table1:** Clinical association between NEURL1B expression and clinicopathological variables in CC patients

Variable	Number	NEURL1B expression		χ^2^-test	Correlation	
		Low	High	P-value	*r*	P-value
Age	279				0.034	0.573
≥ 60	187	88	99	0.573		
< 60	92	40	52			
Gender	279				0.055	0.358
Male	153	74	79	0.358		
Female	126	54	72			
Tumor stage	279				0.012	0.838
T2–4	273	125	148	0.838		
T1	6	3	3			
Lymph metastasis	279				0.047	0.433
Yes	116	50	66	0.433		
No	163	78	85			
Distant metastasis	275				0.071	0.237
Yes	90	36	54	0.237		
No	185	88	97			
Clinical stage	271				0.002	0.980
II–IV	226	105	121	0.980		
I	45	21	24			

^a^Complete data was unavailable in TCGA database

### NEURL1B was an independent prognostic factor in CC

OncoLnc analysis based on TCGA database showed that low expression of NEURL1B predicted a shorter survival time. In addition, univariate Cox regression analysis revealed that lymph metastasis and distant metastasis were important factors to the prognosis of CC (Table 2). However, multivariate Cox regression analysis demonstrated that NEURL1B was an independent prognostic element for CC patients (Table 3).

**Table2:** Univariate analysis of prognostic factors of CC

Variable	OS		
	Hazard ratio	95% CI	*P*-value
Age (≥ 60/< 60)	1.388	(0.772,2.495)	0.273
Gender (male/female)	1.582	(0.934,2.680)	0.088
Tumor size (T2–4/T1)	1.741	(0.240,12.607)	0.583
Lymph metastasis (yes/no)	2.546	(1.515,4.278)	0.000
Distant metastasis (yes/no)	2.863	(1.693,4.843)	0.000
Clinical stage (II–IV/I)	2.984	(0.929,9.589)	0.066
NEURL1B expression (high/low)	0.637	(0.381,1.062)	0.084

**Table 3:** Multivariate analysis of prognostic factors of CC

Variable	OS		
	Hazard ratio	95% CI	P-value
Age, years (≥ 60/< 60)	2.085	(1.043, 4.167)	0.038
Gender (male/female)	1.410	(0.800, 2.485)	0.235
Tumor size (T2–4/T1)	0.269	(0.023, 3.204)	0.299
Lymph metastasis (yes/no)	1.847	(1.012, 3.371)	0.046
Distant metastasis (yes/no)	3.265	(1.761, 6.052)	0.000
Clinical stage (II–IV/I)	2.524	(0.583, 10.927)	0.216
NEURL1B expression (high/low)	0.563	(0.321, 0.988)	0.045

### Hypermethylation of NEURL1B in CC

**Fig. 2:**
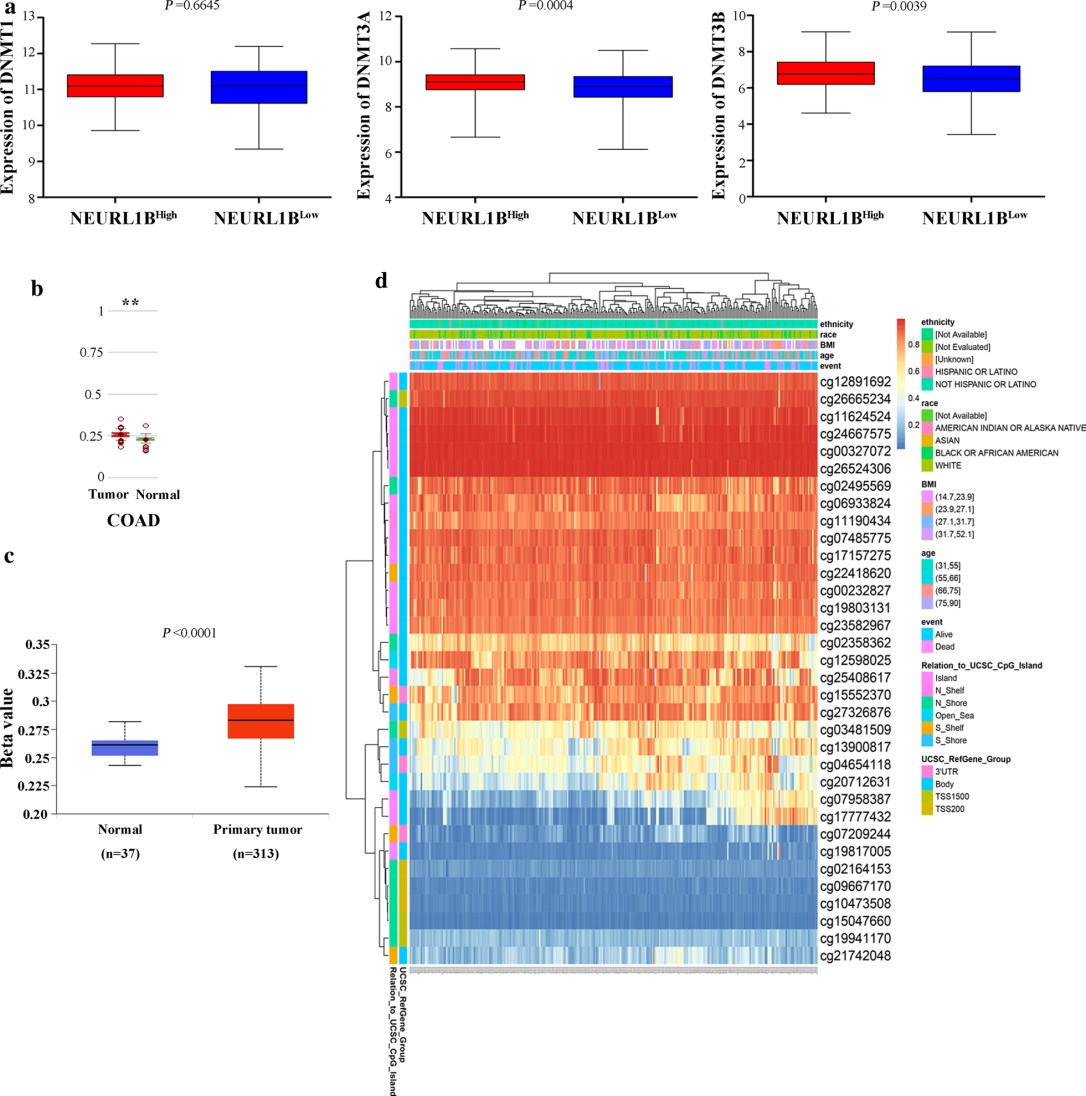
Methylation analysis of NEURL1B. **a** The expression of 3 DNA methyltransferases (DNMT1, DNMT3A and DNMT3B) between NEURL1B^High^ and NEURL1B^Low^ based on TCGA database. **b** Methylated assessment using MethHC. **c** Methylated assessment using UALCAN. **d** NEURL1B-associated differently methylated regions were submitted as heatmap

**Fig. 3:**
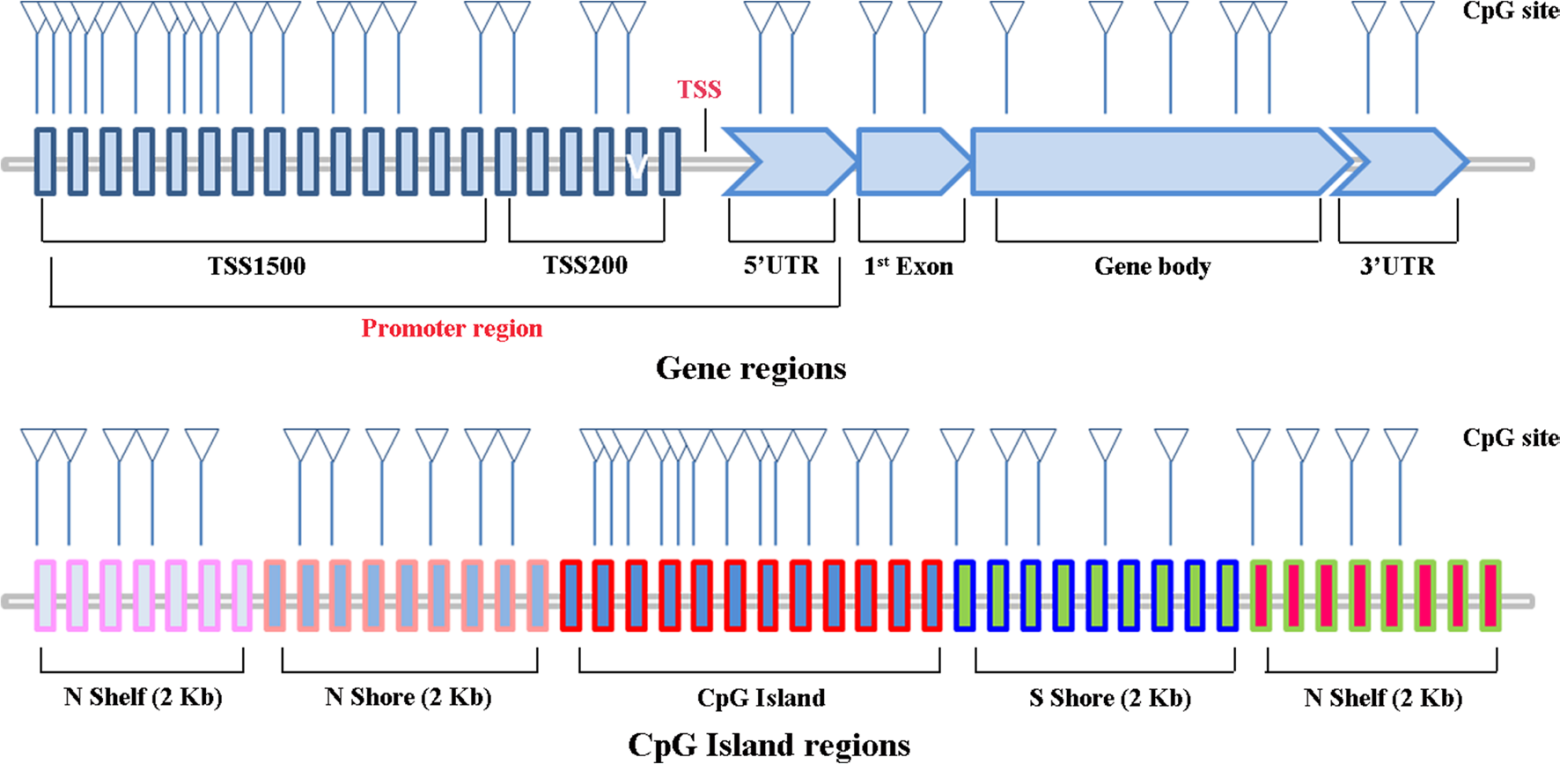
Diagram of gene regions and CpG island regions

To clarify the downregulated mechanism of NEURL1B in CC, we analyzed their methylation status by multiple methods. Expression analysis of 3 DNA methyltransferases showed that NEURL1B^High^ especially co-occuried with higher expression of DNMT3A and DNMT3B (P =0.0004 for DNMT3A and P =0.0039 for DNMT3B, respectively, Fig. 2a). In addition, the analysis from MethHC demonstrated that NEURL1B were significantly higher methylation in CC tissues compared with NC tissues (P < 0.005, Fig. 2b). A similar trend was also found in UALCAN (P < 0.0001, Fig. 2c). Besides, a diagram of gene regions and CpG island regions was drawn (Fig. 3). Position distribution around CpG islands were compared. We found that more hypermethylated sites were around the CpG islands, while more hypomethylated sites lied in open sea regions. Finally, relative position distributions in different locations of gene were also presented. More hypermethylated sites lied on Body regions, while more hypomethylation sitens fell onto TSS1500 and TSS200 regions. Importantly, NEURL1B-associated differently methylated regions were submitted as heatmap(Fig. 2d).

### NEURL1B methylation was associated with clinical staging and prognosis of CC patients

**Fig. 4:**
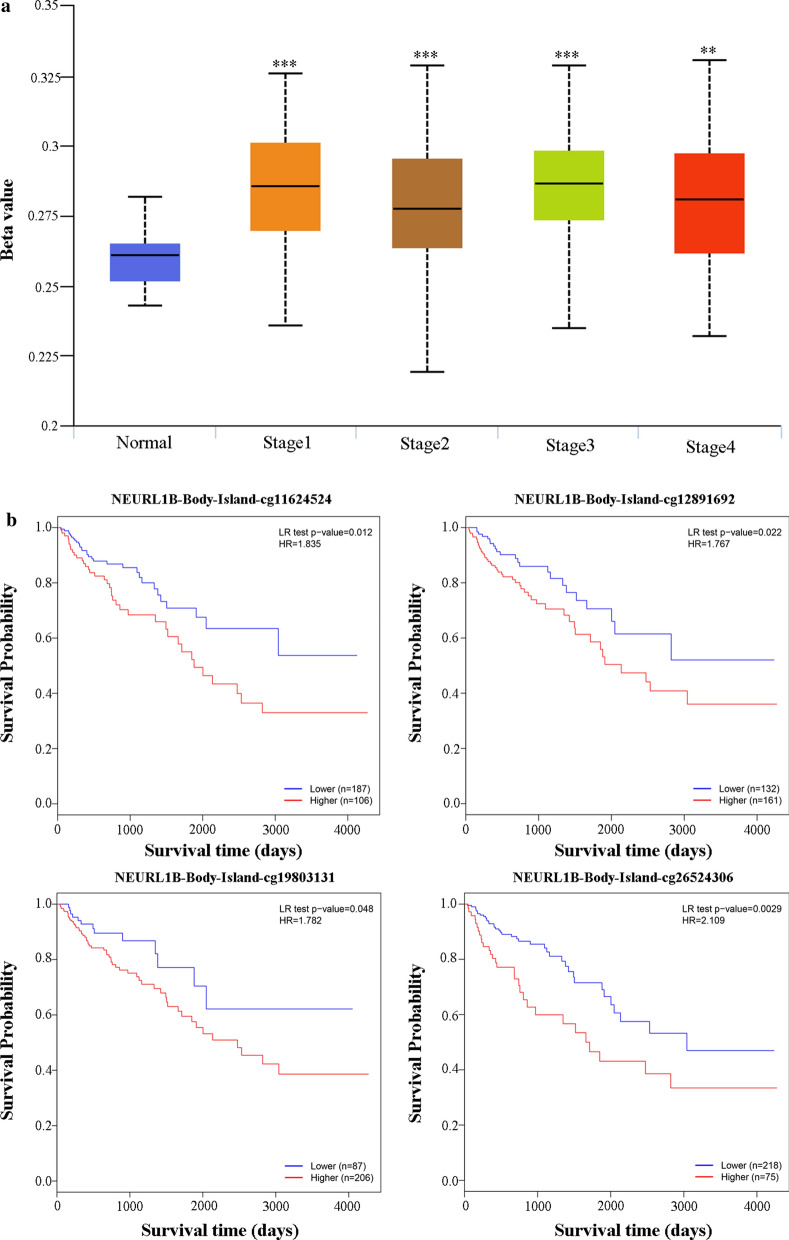
Clinical value of NEURL1B methylation. **a** The association between NEURL1B methylation and clinical staging was shown. ^***^: P < 0.0001; ^**^: P < 0.001. **b** The survival analysis of different methylated regions were investigated using MethSurv tool

UALCAN analysis provided us an important information that NEURL1B methylation exerted a critical role in clinical staging. Compared to NEURL1B normal groups, hypermethylation occurred in whole period, especially stage1 and stage3 (Normal&Stage1, 2.69E−08. Normal&Stage2, 2.02E−05. Normal&Stage3, 5.212E−10. Normal&Stage4, 1.58E−04) (Fig. 4a). Besides, survival analysis of different methylated regions were investigated using MethSurv tool. Compared to NEURL1B^High^ groups in NEURL1B-Body-Island region, NEURL1B^Low^ groups predicted a longer survival time (cg11624524, P = 0.012. cg12891692, P = 0.022. cg19803131, P = 0.048. cg26524306, P = 0.0029 Fig. 4b).

### NEURL1B was associated with multiple activated pathways and proteins

**Fig. 5:**
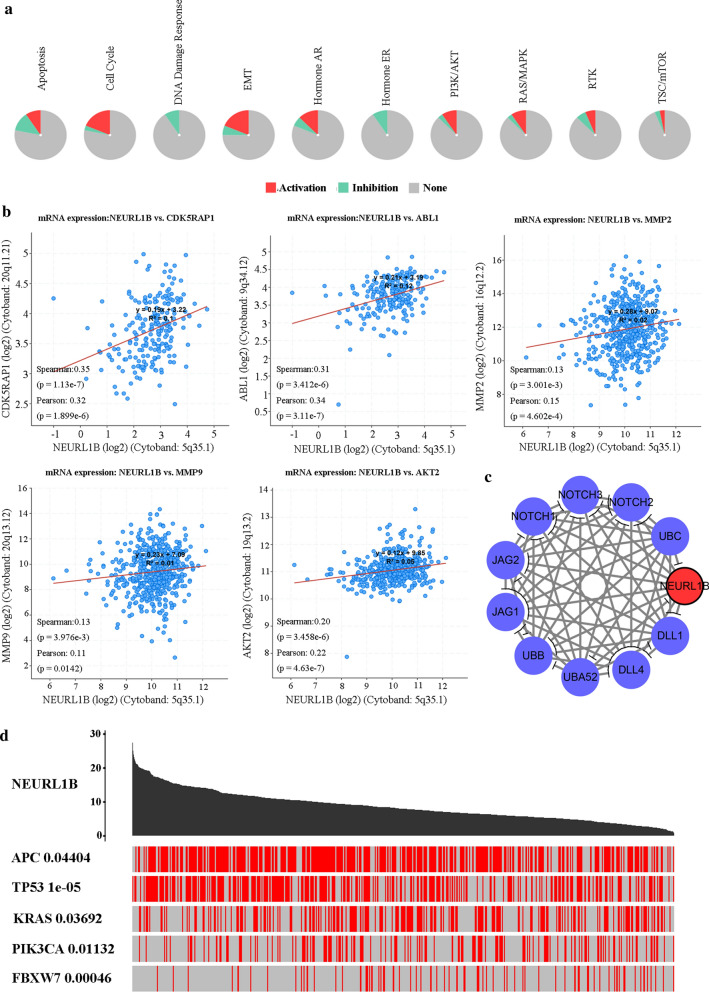
Pathways and PPI network analysis. **a** Pathways analysis using GSCALite. **b** Spearman’s or Pearson’s analysis between NEURL1B expression and some representative molecules in pathways were explored. **c** PPI network analysis based on STRING. **d** A permutatio test p-value of NEURL1B expression between driver mutated and non-mutated samples were examined using TCGAportal

GSCALite analysis on the basis of TCGA database revealed that NEURL1B expression was significantly related to multiple signal pathways, including apoptosis, cell cycle, EMT, PI3K/AKT, and so on (Fig. 5a). Spearman’s or Pearson’s analysis between NEURL1B expression and some representative molecules in pathways also showed a valuable correlation (Fig. 5b). In addition, PPI network analysis revealed NEURL1B could be recognized as a potential ligand to compete with notch receptors (Fig. 5c). Another result from TCGAportal showed that NEURL1B^High^ expression was discovered in APC-mutated samples (P = 0.04404), TP53-mutated samples (P = 1e−05), KRAS-mutated samples (P = 0.03692) and PIK3CA-mutated samples (P = 0.01132). Oppositely, non-FBXW7-mutated samples existed more NEURL1B^High^ expression (P = 0.00046) (Fig. 5d).

### Associations between NEURL1B expression and target-microRNA

**Fig. 6:**
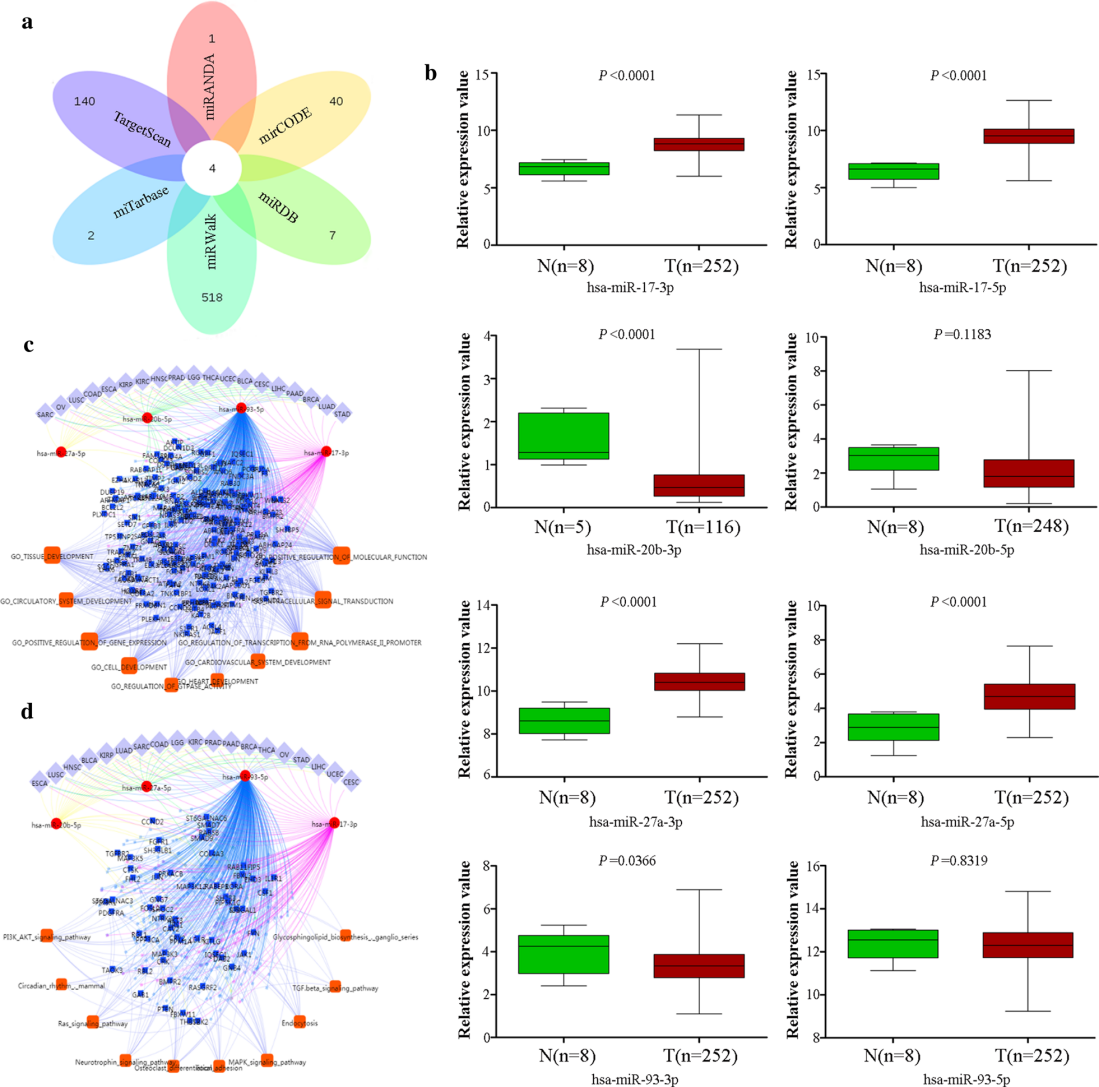
miRNA regulation of NEURL1B. **a** The overlapping NEURL1B-targeted miRNAs. **b** The expression of different miRNAs were detected using TCGA database. **c** miRNAs-dependent GO analysis. **d** miRNAs-dependent signaling pathways analysis

To further explore the other downregulated mechanisms of NEURL1B in CC, target-microRNAs were identified according to various predicted tools. Positively related microRNAs included has-miR-17-3p, has-miR-17-5p, has-miR-20b-3p, has-miR-20b-5p, has-miR-27a-3p, has-miR-27a-5p, has-miR-93-3p and has-miR-93-5p (Fig. 6a). Expression analysis based on TCGA database revealed multiple miRNAs were upregulated in CC tissue (has-miR-17-3p, P < 0.0001. has-miR-17-5p, P < 0.0001. has-miR-27a-3p, P < 0.0001. has-miR-27a-5p, P < 0.0001). However, downregulated miRNAs in CC tissue included has-miR-20b-3p (P <0.0001) and has-miR-93-3p (P = 0.0366) (Fig. 6b). In addition, the biological roles of miRNAs were assessed. GO analysis revealed that the overlapping miRNAs were significantly enriched in positive regulation of gene espression, cellular signal transduction, cell development, positive regulation of molecular function, and so on (Fig. 6c). However, KEGG analysis demonstrated that the intersected miRNAs were mainly focused on PI3K/AKT signaling pathway, Ras signaling pathway, MAPK signaling pathway, TGF-beta signaling pathway, and so on (Fig. 6d).

### NEURL1B was a new diagnostic biomarker

**Fig. 7:**
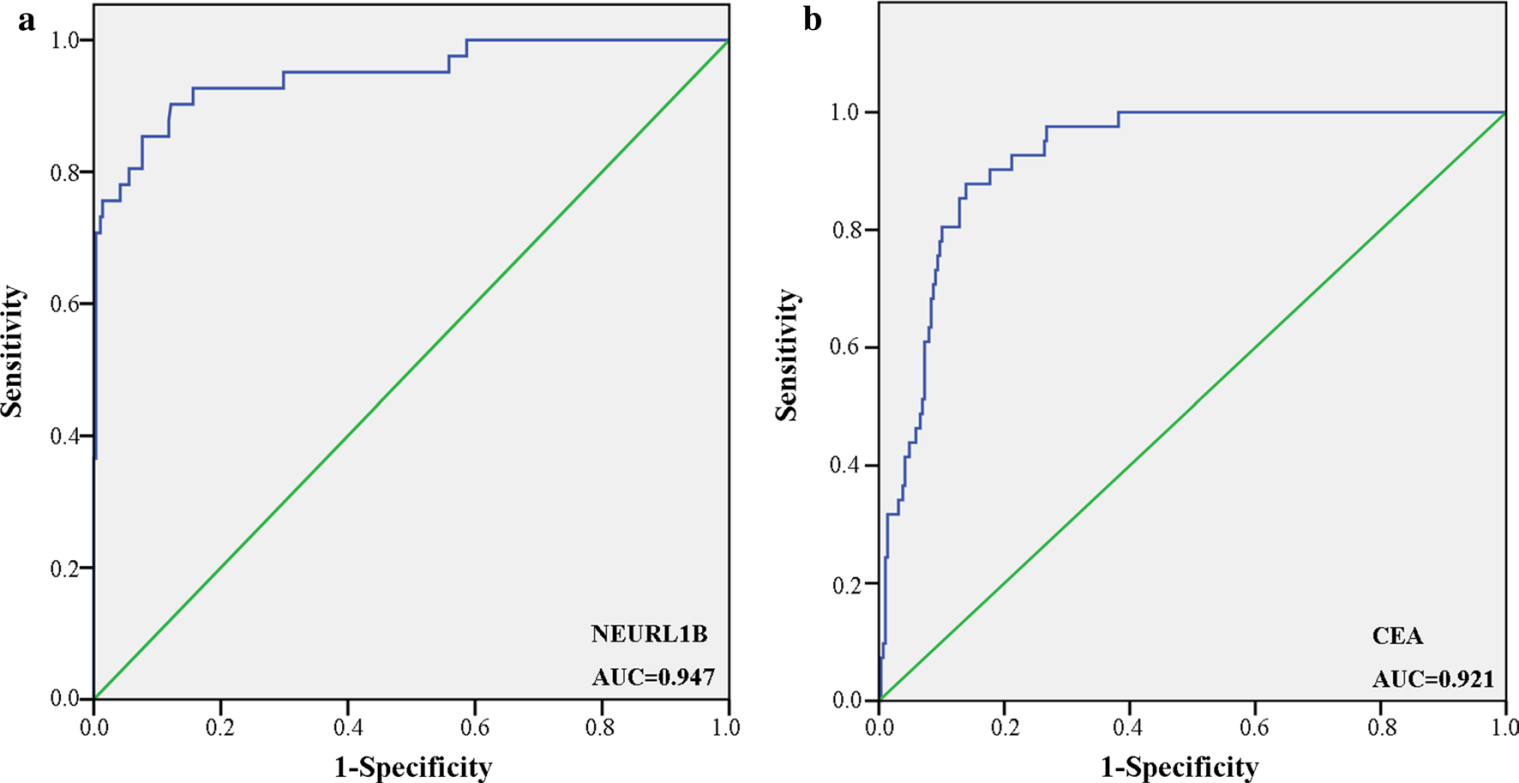
Diagnostic value of NEURL1B. **a** ROC curve analysis of NEURL1B. **b** ROC curve analysis of CEA

Carcinoembryonic antigen (CEA) is a common tumor marker for the diagnosis of colorectal cancer in clinical. Compared to CEA (AUC=0.921), NEURL1B showed a more area under the curve (AUC). An AUC value of 0.947 indicated a strong diagnostic ability of NEURL1B for CC (Fig. 7).

## Discussion

Human Neuralized (hNeur), one of the E3 ubiquitin ligases, included two homologs: hNeur1 (NEURL1) and hNeur2 (NEURL1B) [23]. Until now, hNeur1 was considered to be related to spermatogenesis and mammary gland maturation [24]. Besides, hNeur1 contributed an important function in the nucleus based on a shuttle circulation between the nucleus and cytoplasm. In addition, another function as a transcriptional repressor was also cinfirmed in vitro [25]. However, the role of NEURL1B in human has not been studied. To further explore the characteristic of NEURL1B in CC, its expression patterns, prognosis analysis, methylation mode and target-miRNAs analysis were described in our study.

NEURL1B is located on chromosome 5q31 and consists of five exons. The full-length protein comprises of 555 amino acids. There are 3 crucial domains in the NEURL1B protein, including the RING Zn-finger (RZD) domain and two Neuralized Homology Repeat (NHR) domains [26]. It was confirmed that NEURL1B was mainly localized in cytoplasm and peak expression was focusd in embryogenesis [26]. In our present study, a new characteristic of tumor suppressor gene for NEURL1B was shown in CC. However, a differently compared result were obtained when 13 matched pairs of clinical samples were analysed. Besides, χ^2^-test analysis also revealed that NEURL1B expression was not related to multiple clinicopathological variables, including age, gender, tumor stage, lymph metastasis, distant metastasis and clinical stage. The possible reasons were considered as follow: (1) limited cases based on TCGA database and clinical samples were included in the present study, more clinical samples need to be collected to evaluate the role of NEURL1B in future. (2) Multiple mixed facters were involved in the occurrence and progress of tumor. χ^2^-test was used to assess the correlation between two clinicopathological characteristics, some other risk facters couldn’t be excluded in our study.

Aberrant methylation of gene promoter is an early and frequent event during the initial stage of tumor, which is closely related to tumor occurrence, development and prognosis. DNA methylation, as an important part of epigenetics, is more widely present in almost all tumors than other types of DNA molecule abnormalities, including mutations, microsatellite sequence change, abnormal gene amplification and chromosomal abnormalities, and so on [27]. In addition, DNA methylation plays an important role in regulating gene expression, especially for tumor suppressor genes [27]. Therefore, these frequent molecular events can be used as a marker for early diagnosis and prognostic evaluation of CC. In the current study, we analyzed promoter methylation of NEURL1B gene using multiple bioinformatic tools based on TCGA database. Consistent results showed that NEURL1B was hypermethylated in CC tissues. Combined with DNA methyltransferases analysis (DNMT1, DNMT3A and DMNT3B), we found NEURL1B^High^ group obviously co-occured with higher expression of DNMT3A and DNMT3B, which promoted us to understand the downregulated expression of NEURL1B in CC. In line with our finding was a reported mechanisms that the DNA methylation have a notable influences on gene expression [28]. Besides, inspired by the positive correlation between differently methylated sites and prognosis of CC patients, as well as the associations between NEURL1B methylation and clinical stages, suggesting that this epigenetic modification might be a potentially increased risk of colon cancer-related death. Simultaneously, methylation-associated inactivation of NEURL1B might be designed as a target in future investigation. However, bioinformatics analysis are only a predictive tools based on TCGA database, which could provide a new understanding for our next research, More in vivo and in vitro studies need to be done to confirm the status and level of NEURL1B methylation.

Considering the fact that the pathogenesis of NEURL1B in CC remains unclear, we firstly analysed NEURL1B-related pathways. The results showed that activated signaling pathways were mainly involved in multiple biological processes, including cell cycle regulation, apoptosis, and metastasis, which was consistent with our χ^2^-test analysis. In addition, we investigated the role of some representative molecules assocoated with NEURL1B expression. CDK5RAP1 encodes a regulator of cyclin-dependent kinase 5 activity, which has also been reported to have a function as an inhibitor of cell apoptosis [29]. ABL1 is a protooncogene that encodes a protein tyrosine kinase involved in a variety of cellular processes, including cell division, adhesion, differentiation, and response to stress [30]. Proteins of the matrix metalloproteinase (MMP) family are involved in the breakdown of extracellular matrix in normal physiological processes, such as embryonic development, reproduction, and tissue remodeling, as well as in disease processes, such as arthritis and metastasis [31]. AKT serine/threonine kinase 2 (AKT2) is a putative oncogene encoding a protein belonging to a subfamily of serine/threonine kinases containing SH2-like (Src homology 2-like) domains. The encoded protein is a general protein kinase capable of phophorylating several known proteins [32]. All analysis based on Spearman’s or Pearman’ correlation revealed that a weak binding existed between NEURL1B expression and these key molecules, suggesting that NEURL1B may function by interacting with other proteins in the pathway. Therefore, we further explored protein-protein interaction (PPI) network of NEURL1B according to STRING. A new function was visible that NEURL1B could interact with Notch ligands, which might activate the Notch pathway. A possible reason was speculated based on the precious analysis, we found that NEURL1B might be a tumor suppressor gene in colon cancer, whose decrease could further reduce this antagonism. In future, we will also design relative analysis to explore the specific mechanism in colon cancer. It is consistent with Rullinkov’s reports [33]. Besides, a significant association between NEURL1B expression and high frequency mutant gene was shown, suggesting that NEURL1B might be involved in the initiation and development of CC. More experiments in vitro and in vivo need to be done to confirm our hypothesis.

To further clarify whether NEURL1B had other downregulated mechanisms in CC. Multiple website tools were executed to identify NEURL1B-target miRNAs, 4 overlapping goals were obtained. Encouraged by the aberrant expression of miRNAs, the result revealed miR-17 and miR-27a were overexpressed in CC tissues, which might be another explanation for downregulated expression of NEURL1B. In addition, these miRNAs have been found to have important tumorigenesis values in previous studies. miR-17-5p was abnormally expressed in various tumor types and its overexpression could predict a shorter survival times in lung cancer [34]. Simultaneously, miR-17 was also proved to induce EMT process by regulating CYP7B1 expression in colon cancer [35]. miR-27a was highly expressed in gastric cancer tissues and cells, and it might promote cell proliferation, migration and invasion by targeting SFRP1 via the activation of Wnt/β-catenin signaling pathway [36]. All these aberrant regulation of miRNAs would help us to understand the poor prognosis of NEURL1B^Low^ group.

GO and pathway analysis were carried out to elucidate the biological functions of microRNAs. We found that multiple pathways were activated and these pathways have been found to be involved in the proliferation, migration and invasion of tumor. To be excited, there existing be an significant intersection between miRNAs-target pathways and NEURL1B-target pathways, suggesting that miR-17 and miR-27a might promote tumor cell malignant property by targeting NEURL1B via the activation of PI3K/AKT signaling pathway. Without a doubt, The bioinformatic analysis based on multiple database only provides us an open platform. More unknown functions and deep investigation need to be done to assess the role of NEURL1B in CC.

## Conclusions

In summary, we analyzed the diagnostic and prognostic significance of NEURL1B on the basis of TCGA database in CC, and demonstrated that aberrant methylation and target-miRNAs were strongly associated with downregulation of NEURL1B. More importantly, hypermethylation of NEURL1B-Body-Island was closely ralated to adverse outcome of CC patients. Thus, NEURL1B may be served as potential biomarkers for early diagnosis and prognostic evaluation in CC.

## Data Availability

The datasets used during the current study are available from the corresponding author on reasonable request.

## Abbreviations

CC: Colon cancer
NC: Normal colon
FC: Fold change
OS: Overall survival
ROC: Receiver operating characteristic
TCGA: The Cancer Genome Atlas
CEA: Carcinoembryonic antigen
AUC: An area under curve
DEGs: Differentially expressed genes
